# From Survival to Glamour: Motivations for Engaging in Transactional Sex and Relationships Among Adolescent Girls and Young Women in South Africa

**DOI:** 10.1007/s10461-021-03291-z

**Published:** 2021-05-05

**Authors:** Zoe Duby, Kim Jonas, Tracy McClinton Appollis, Kealeboga Maruping, Lieve Vanleeuw, Caroline Kuo, Catherine Mathews

**Affiliations:** 1 Health Systems Research Unit, South African Medical Research Council, Francie van Zijl Drive, Parow Valley, Tygerberg, Cape Town, South Africa; 2 Division of Social and Behavioural Sciences in the School of Public Health and Family Medicine, University of Cape Town, Cape Town, South Africa; 3 Department of Psychiatry and Mental Health, University of Cape Town, Cape Town, South Africa; 4 Department of Behavioral and Social Sciences, Providence/Boston Center for AIDS Research, Brown University School of Public Health, Rhode Island, USA

**Keywords:** Transactional sex, Transactional relationships, Adolescent girls and young women, South Africa

## Abstract

We explored transactional sex and relationships (TSR) among South African adolescent girls and young women (AGYW) using (1) survey data from 4,399 AGYW aged 15–24 years, and (2) qualitative data from 237 AGYW and 38 male peers. Ten percent of sexually active AGYW reported having ever had transactional sex; 14% reported having stayed in a relationship for money or material items. Factors associated with higher reporting of TSR included HIV positivity, higher food insecurity, and alcohol use. Those AGYW who were between the ages of 20–24 years (OR: 1.0; 95% CI: 0.81–1.24), had a sexual partner older than her by 5 years or more (OR: 1.89; 95% CI: 1.58–2.26), and had a transactional relationship in the past (OR: 61.1; 95% CI: 47.37–78.76) were more likely to report having transactional sex. AGYW qualitative narratives included both assertions of agency in choosing to engage in TSR, and power inequities resulting in condomless sex. Our findings can inform interventions to addressing transactional sex and relationships, critical to South Africa’s HIV response.

## Introduction

The association of transactional sex and HIV risk in adolescent girls and young women (AGYW) in sub–Saharan Africa has been clearly demonstrated, with evidence showing increased susceptibility to HIV infection among AGYW who engage in transactional sex [1–3]. AGYW who engage in transactional sex are more likely to experience poor sexual and reproductive health outcomes, including unintended pregnancies, unsafe abortions, sexually transmitted infections (including HIV), and sexual coercion [4]. Among the reasons that transactional sex is associated with greater risk of contracting HIV are compromised gendered power relations and the likelihood of having multiple partners [5].

Transactional sex is usually defined as a specific set of behaviours, labels and identities, distinct from commercial sex work, and characterised by the exchange of financial or material support, which occurs outside of the context of marriages, or ‘formal’ sex work [3, 6]. The key feature used to distinguish transactional sex and transactional relationships (TSR) from other non–marital romantic/ sexual relationships, is that they are not only characterised by material exchange, but motivated by it [7]. An additional feature is that TSR are differentiated from, and judged by those who participate in the exchange to be more morally acceptable than formal sex work, primarily based on the fact that the exchange occurs within the context of a relationship (no matter how temporary or ambiguous its nature) [1, 8]. However the distinction between transactional sex, sex work, and romantic/sexual relationships is challenging, as the boundaries are very blurred [9].

Evidence suggests a positive association between age–disparate, or age-asymmetrical, partnerships and HIV–infection risk among young women in South Africa [10, 11]. Although AGYW may exercise some agency in choosing to engage in age-disparate partnerships that are transactional in nature, unequal gendered power dynamics are heightened with age-disparity, exacerbating AGYW HIV risk by negatively impacting their ability to negotiate condom use [12, 13].

Motivations for engaging in TSR are situated within complex sexual economies, and go beyond basic survival or subsistence needs in circumstances of poverty [8, 14]. Qualitative research has described entrenched social norms dictating that material or financial goods received from a partner must be reciprocated with sex, framing the narrative around transactional sex to include both survival and acquisition of basic needs, as well as for gain in social status [15, 16]. The very expectation of reciprocity, and the act of receiving material benefits, enhances the vulnerability of the receiver, thereby limiting their agency [16]. Motivations for TSR include seeking peer approval and a sense of belonging, the desire for a ‘modern’ lifestyle as dictated by society, the media, and to attain financial independence, prestige and social standing [3, 8]. While it is evident that in certain cases, TSR is driven by structural factors, including poverty, gender inequality, and lack of education, it is also driven by a number of psychosocial factors, including societal/familial/peer pressure, aspirations for social mobility, and material consumer goods, as well as romantic notions of love and security [1].

Given the slipperiness of definitions and identities relating to transactional sex, transactional relationships, sex work and romantic/sexual relationships, it is important to understand how young South Africans define and conceptualise sexual transactions and relationship dynamics that are informed by gendered power and sexual norms [9]. Most of the studies exploring attitudes related to TSR in South Africa have not included male perspectives, and there has been a call for research which examines men and boys’ perspectives and opinions on transactional sexual encounters [14]. Additionally, many of the studies that have examined TSR in South Africa have focused on one geographic area. Our study includes data from a range of urban, semi–urban, and rural sites across six provinces of South Africa. Combining quantitative data on AGYW reporting of having engaged in TSR, alongside qualitive narratives and perspectives on TSR from AGYW and their male peers, we are able to deepen understanding of these complex risk behaviours, and the factors that influence AGYW decision making around TSR, helping to inform interventions that seek to address sexual and reproductive health challenges among AGYW in South Africa.

## Methods

The data presented in this paper comprise quantitative and qualitative data from a larger evaluation study of a combination HIV prevention intervention for AGYW in South Africa (https://www.samrc.ac.za/intramural–research–units/HealthSystems–HERStory), which was funded by the Global Fund. The examination of TSR was not initially a key focus of the larger study, but emerged as important in understanding the broader context of HIV risk behaviours. For the purposes of this study we use the term Adolescent Girls and Young Women (AGYW) to refer to females between the ages of 15 and 24 years. In line with the World Health Organization’s definitions of “early adolescence” (10–14), “late adolescence” (15–19) and “post-adolescence” (20–24) (www.who.int/health-topics/adolescent-health), The Global Fund policy literature refers to AGYW aged 15–24 comprising of “adolescent girls” (ages 15–19), and “young women” (ages 20–24) (www.theglobalfund.org).

### Survey with AGYW

Quantitative data presented in this paper comprises findings from a cross–sectional survey conducted 2017–2018 among 4,399 AGYW in six South African districts in which the combination HIV prevention intervention was implemented: City of Cape Town (Western Cape), Ehlanzeni (Mpumalanga), O.R. Tambo (Eastern Cape), Tshwane (Gauteng), and King Cetshwayo and Zululand (KwaZulu–Natal). A representative sample of households in the intervention areas was selected for inclusion, and all AGYW aged between 15 and 24 years in the sampled households were invited to participate. The sample realisation for the survey was 61%, ranging from 33% in Cape Town to 78% in Zululand.

The survey included questions relating to TSR; we report on the following multi–choice items where respondents could select more than one option: (1) Have you ever given oral, anal, or vaginal sex to someone because you expected to get or got any of these things? (2) In the past 12 months have you started or stayed in a relationship with a man or boy so that you could receive any of the following? Answer options were: Money; Transport; Food for myself and/or my family; Clothes or shoes; Shelter; School fees/school uniforms; Airtime; Cellphone; Items for children or family; Cosmetics; I have not done this; I prefer not to say; Other. In addition, AGYW were asked to report on economic variables including whether: (1) in the past month, participant or household member went a day and night without eating because of lack of food; and (2) household depends on child support grant, foster care grant, disability grant, or pension.

The survey was conducted via electronic questionnaires, administered by a fieldworker using a tablet. Sections of the questionnaire with sensitive questions, including those related to transactional sex, were completed by the participants themselves to diminish social desirability bias that might otherwise affect the quality of data (Table 1). The fieldworker read each question to the participant and allowed the participant to enter her responses in the tablet privately.

**Table 1:** HERStory qualitative study sample by site

Province	Western Cape (WC)	KwaZulu–Natal (KZN)	Mpumalanga (MPU)	North West (NW)	Eastern Cape (EC)	
District	City of Cape Town	King Cetshwayo	Gert Sibande	Bojanala	Nelson Mandela Bay	
Characteristic	Urban	Rural	Semi–urban	Semi–urban	Urban	

Sample group	N	N	N	N	N	Total n
AGYW aged 15–19 years	52	28	33	26	38	177
AGYW aged 20–24 years	11	22	8	9	10	60
Total AGYW aged 15–24 years	**63**	**50**	**41**	**35**	**48**	**237**
Male peers aged 18–24 years	7	8	7	2	14	**38**

The HIV status of participants (as presented in Tables 2 and 3) was determined using laboratory analysed blood samples. The samples were tested with Genscreen Bio-Rad HIV1/2 Combi Assay and any reactive result was confirmed by a second 4th Generation test (Roche HIV1/2 COMBI COBAS E411). All positive specimens were confirmed for HIV-1 infection by Western blot (GS HIV-1 Western Blot, Bio-Rad Laboratories, Redmond, WA 98,052, USA). Participants were contacted by study team members, and reminded to collect test results at the clinic they had indicated as their preferred/nearby clinic. For those participants who missed clinic appointments, or needed additional support, study staff would follow up, and if necessary be present at the clinic to assist the AGYW to link with the clinic staff to receive results.

**Table 2:** Transactional sex among sexually active adolescent girls and young women aged 15–24 years (n = 3009) in the HERStory study

Variable	Transactional sex										
	Overall			Did not report transactional sex			Reported transactional sex				
	n	%	95% CI	n	%	95% CI	n	%	95% CI	Chi Square ( χ^2^) | p-value	95% CI
Age group											
15–19	1300	43.3	41.8–44.8	1151	88.8	87.2–90.3	149	11.2	9.7–12.8	3.02 | 0.08	− 0.20–3.56
20–24	1709	56.7	55.2–58.2	1481	87.1	85.8–88.4	228	12.9	11.6–14.2		
HIV Status†											
Positive	499	15.9	14.8–17.0	414	83.7	81.1–86.1	85	16.3	13.9–18.9	14.31 | < 0.01	− 7.50–-2.35
Negative	2509	84.1	83.0–85.2	2217	88.6	87.5–89.7	292	11.4	10.3–12.5		
Reported higher food insecurity											
No	2431	80.6	79.2–82.0	2163	89.3	88.4–90.2	268	10.7	9.8–11.6	25.76 | 0.00	4.73–10.38
Yes	578	19.4	18.0–20.8	469	81.8	78.7–84.5	109	18.2	15.5–21.3		
Household social grant dependency											
No	1572	54.3	52.5–56.2	1384	88.5	87.2–89.7	188	11.5	10.3–12.8	1.87 | 0.17	− 0.56–3.27
Yes	1437	45.7	43.8–47.5	1248	87.1	85.5–88.6	189	12.9	11.4–14.5		
Had high alcohol use (Audit-C score 2 or higher)											
No	2082	67.5	65.9–69.1	1856	89.5	88.3–90.7	226	10.5	9.3–11.7	23.53 | 0.00	3.10–7.22
Yes	927	32.5	30.9–34.1	776	84.4	82.5–86.1	151	15.6	13.9–17.5		
Partner older than 5 years											
No	1987	66.2	64.8–67.6	1792	90.6	89.4–91.6	195	9.4	8.4–10.6	55.42 | 0.00	
Yes	1022	33.8	32.3–35.2	840	82.5	80.6–84.3	182	17.4	15.7–19.4

**Table 3:** Reporting of staying in a relationship to receive money or goods among sexually active AGYW (n = 3009)

Variable	Transactional relationship									Chi square (χ^2^) | p-value	95% CI
	Overall			Did not report a transactional relationship			Reported a transactional relationship				
	n	%	95% CI	n	%	95% CI	n	%	95% CI		
Age group											
15–19	1300	43.3	41.8–44.8	1130	87.2	85.6–88.8	170	12.8	11.2–14.4	2. 318 | 0.1264	− 0.45–3.69
20–24	1709	56.7	55.2–58.2	1450	85.6	84.1–87.0	259	14.4	13.0–15.9		
In school											
No	1669	56.0	54.5–57.6	1418	85.6	84.1–86.9	251	14.4	13.1–15.9	2.73 | 0.10	
Yes	1340	44.0	42.4–45.5	1162	87.3	85.6–88.8	178	12.7	11.1–14.4		
HIV Status †											
Positive	499	15.9	14.8–17.0	404	81.8	78.9–84.4	95	18.2	15.6–21.1	14.41 | <0.01	− 8.19– − 2.63
Negative	2509	84.1	83.0–85.2	2175	87.2	86.0–88.3	334	12.8	11.7–14.0		
Reported higher food insecurity											
No	2431	80.6	79.2–82.0	2135	88.3	87.2–89.3	296	11.7	10.7–12.8	36.66 | 0.00	7.09–13.42
Yes	578	19.4	18.0–20.8	445	78.1	74.8–81.1	133	21.9	18.9–25.2		
Household social grant dependency											
No	1572	54.3	52.5–56.2	1360	87.2	85.7–88.5	212	12.8	11.5–14.3	2.93 | 0.08	− 0.25–3.92
Yes	1437	45.7	43.8–47.5	1220	85.3	83.6–87.0	217	14.7	13.0–16.4		
Had high alcohol use (Audit-C score 2 or higher)											
No	2082	67.5	65.9–69.1	1821	87.9	86.5–89.2	261	12.1	10.8–13.5	18.19 | 0.00	2.58–6.95
Yes	927	32.5	30.9–34.1	759	83.1	81.2–84.9	168	16.9	15.1–18.8		
Partner older than 5 years											
No	1987	66.2	64.8–67.6	1764	89.2	87.9–90.4	223	10.8	9.6–12.1	55.8 | 0.00	
Yes	1022	33.8	32.2–35.2	816	80.6	78.6–82.5	206	19.4	17.5–21.4		

We used a brief version of the twelve-item Alcohol Use Disorders Identification Test (AUDIT), namely AUDIT-C, to describe the prevalence of hazardous drinking among AGYW (Cronbach’s Alpha: 0.79). AUDIT-C comprises the first three items of AUDIT which measure self-reported alcohol consumption, and which has been found to be comparable to AUDIT, including among a South African population [17]. A participant’s AUDIT-C score can range from 0 to 12. Informed by the recommendation emanating from the South African study [17], we used a cut-off score of greater than or equal to 2 to indicate hazardous drinking.

### Qualitative Study Component Including AGYW and Young Men

Combined with survey data, this analysis included data from the qualitative study component conducted August 2018 and March 2019 in five South African districts: City of Cape Town, Western Cape (WC); King Cetshwayo, KwaZulu–Natal (KZN); Gert Sibande, Mpumalanga (MPU); Bojanala, North West (NW); and Nelson Mandela Bay, Eastern Cape (EC). Participants in the qualitative study component were recruited independently from the quantitative study component, and were not necessarily the same AGYW who had participated in the survey, or that had reported transactional sex in the survey. Male peer respondents aged 18–24 years, were not necessarily sexual partners of the AGYW respondents, and were recruited in schools, and from the communities in which the intervention was being implemented.

Qualitative methods included 63 in–depth interviews (IDIs) and 24 focus group discussions (FGDs) with 237 AGYW aged 15–24 years, as well as six FGDs with 38 young men. In-depth interviews conducted with individuals lasted approximately 20–40 min. Focus group discussions, each with 6–10 participants from the same sample group, lasted approximately 40–90 min. IDIs and FGDs were conducted by experienced, trained female researchers, in English, isiZulu, isiXhosa, seTswana, or siSwati, using semi–structured topic guides with open–ended questions and probes for potential additional issues. Discussion topics for both male and female respondents included perceptions of sexual and romantic relationship norms and ideals, gendered power and sexual decision making. No specific questions pertaining to TSR were included in the topic guides; however, questions pertaining to sexual and romantic relationships sparked discussions on the topic. A brief demographic questionnaire was also administered.

### Quantitative Data Analysis

Quantitative data were analysed using Stata/SE 14.2 (StataCorp 2015). Descriptive summary statistics were performed to provide frequency tables, and percentages of the participants’ responses to the key variables. Pearson’s chi–square tests were used to describe the association between reports of higher food insecurity and household dependency on social grants and transactional sex/transactional relationships, with significance set at p value equals to or less than 0.05 (p ≤ 0.05). Finally, we conducted a multiple logistic regression analysis to identify the factors strongly associated with transactional sex among the AGYW in this study. The significance was set at 95% confidence interval (95% CI) with the associated p-value equal to or less than 0.05 (p ≤ 0.05). Data were weighted due to the complex sampling design and sample weights were based on the probability of sampling small area layers (SALs, the primary sampling unit) in each district. The total weighted sample size of AGYW aged 15–24 years who participated in the survey was 7,237. The proportions that are presented are weighted, in order to be considered to be representative of the population, which increases the generalizability of the quantitative results. We also report the unweighted frequencies. The results are presented in tables and accompany the qualitative thematic areas.

### Qualitative Data Analysis

Audio recordings of IDIs and FGDs were transcribed verbatim into the original language, reviewed by the interviewer for fidelity to the interview, translated into English and reviewed again to ensure correct interpretation and the accuracy of translations. Analysis followed a thematic cyclical approach, in which a pre–determined deductive codebook was developed, based on the research objectives and the topic guides. Codes underwent inductive development and refinement, and were entered into NVivo 12 software to assist with the labelling and organisation of data [18–20]. Collaborative interpretation by the research team included data immersion and familiarisation, repeated readings of transcripts, pattern finding, and documentation of theoretical and reflective thoughts. Weekly research meetings were held throughout the data collection and analysis phases allowing for team debriefing and examination of emergent themes, and evolving engagement with and interpretation of the data. In addition, feedback workshops were held with AGYW respondents to confirm the research team’s accurate and appropriate interpretation of the data.

Quantitative and qualitative findings are presented, with comparisons made between provinces, where appropriate or noteworthy. Qualitative findings are arranged into key thematic areas that emerged during analysis and combined with related quantitative findings. Illustrative quotations are excerpts from English transcripts or translations; in brackets are details of the respondents’ site and sample group.

## Findings

### Quantitative Findings

Of the 4,399 AGYW aged 15–24 years surveyed, most (69.2%, n = 3,009/4,339) reported having ever had “sex”, defined as “when the penis enters the vagina or anus/bum”, 52.8% (n = 1300/2515) in the age group 15–19 years and 90.5% (n = 1709/1884) in the age group 20–24 years.

#### Transactional Sex and Transactional Relationships

Among AGYW who had ever had sex, 12.1% (n = 377) reported ever having engaged in transactional sex (had oral, anal or vaginal sex with someone in the expectation of receiving money or goods) (Table 2): 12.9% of those in the 20–24 year age group, and 11.2% in the 15–19 year age group (χ^2^ = 3.02, p = 0.08). Among HIV positive sexually active AGYW, 16.3% reported transactional sex, while 11.4% among HIV negative sexually active AGYW reported it (χ^2^ = 14.3, p < 0.01) (Table 2).

Among sexually active AGYW, 13.7% (n = 429) reported they had stayed in a relationship for money or goods (transactional relationships). Disaggregated by age, 12.8% of those aged 15–19, and 14.4% of those aged 20–24 (χ^2^ = 2.32, p = 0.13) (Table 3), engaged in a transactional relationship. Among HIV positive AGYW, 18.2% reported transactional relationships, whereas only 12.8% of HIV negative AGYW reported them (χ^2^ = 14.4, p < 0.01) (Table 3). There were approximately five fewer cases of transactional relationships per 100 AGYW among those who were HIV negative.

Reporting of transactional sex was higher among AGYW who had high alcohol use (15.6%), than those who did not (10.5%, χ^2^ = 23.5, p < 0.01) (Table 2). Similarly, reporting of engaging in transactional relationships was higher among those AGYW categorised as having high alcohol use (16.9% compared to 12.1%), (χ^2^ = 18.2, p < 0.01) (Table 3).

For reporting on commodities exchanged for sex, 6.7% of AGYW who had ever had sex reported to have ever given oral, anal, or vaginal sex to someone in exchange for money (7.8% amongst AGYW aged 20–24 years (χ^2^ = 244.7, < 0.01), and 5.3% amongst those aged 15–19 years, (χ^2^ = 96.6, < 0.01). Of the sexually active AGYW, 8.7% reported having started or stayed in a relationship with a man/boy (in past 12 months) in order to receive money (7.4% in the 15–19 age group (χ^2^ = 136.82, < 0.01) and 9.8% in the 20–24 years age group (χ^2^ = 226.73, < 0.01). Other commodities traded in transactional relationships by sexually active AGYW included airtime (3.1%), cosmetics/makeup (2.4%), and clothes/shoes (2.3%) (Table 4).

**Table 4:** Commodities traded for transactional sex and transactional relationships among sexually active AGYW (n = 3009)

Variable	Ever GIVEN oral, anal, or vaginal sex to someone in exchange for:				Started/stayed in a relationship with a man/boy (in past 12 months) so you could receive:			
	n	%	Chi Square (χ^2^) | p-value	95% CI	n	%	Chi square (χ^2^) | p-value	95% CI
Money								
Total 15–24 (n = 3009)	215	6.7	342.4 | < 0.001	6.07–7.47	276	8.7	325.12 | < 0.001	7.86–9.71
15–19 (n = 2515)	73	5.3	96.6 | < 0.001	4.34–6.49	100	7.4	136.82 | < 0.001	6.19–8.71
20–24 (n = 1884)	14	7.8	244.7 | < 0.001	6.89–8.87	176	9.8	226.73 | < 0.001	8.60–11.15
Transport								
Total 15–24	38	1.2	64.5 | < 0.001	0.92–1.49	43	1.4	63.23 | < 0.001	1.09–1.78
15–19	12	0.8	21.1 | < 0.001	0.53–1.24	14	1.1	23.22 | < 0.001	0.71–1.60
20–24	26	1.5	40.9 | < 0.001	1.06–1.98	29	1.7	38.67 | < 0.001	1.21–2.26
Food for myself and/or my family								
Total 15–24	55	1.7	74.3 | < 0.001	1.38–2.18	65	2.1	97.84 | < 0.001	1.75–2.61
15–19	26	1.9	33.7 | < 0.001	1.36–2.71	22	1.7	26.29 | < 0.001	1.18–2.56
20–24	29	1.6	42.3 | < 0.001	1.78–2.15	43	2.4	67.54 | < 0.001	1.91–3.10
Clothes or shoes								
Total 15–24	47	1.4	80. 1 | < 0.001	1.13–1.74	72	2.3	122. 95 | < 0.001	1.90–2.71
15–19	16	1.1	29.3 | < 0.001	0.77–1.59	24	1.7	39.89 | < 0.001	1.25–2.35
20–24	31	1.6	56.4 | < 0.001	1.26–2.11	48	2.7	78.40 | < 0.001	2.16–3.35
Shelter								
Total 15–24	9	0.2	14.4 | 0.002	0.15–0.42	15	0.5	26.38 | < 0.001	0.33–0.70
15–19	7	0.4	9.9 | 0.002	0.23–0.79	8	0.6	11.57 | 0.001	0.33–1.03
20–24	2	0.1	3.6 | 0.060	0.04–0.32	7	0.4	12.79 | 0.000	0.23–0.70
School fees/school uniforms								
Total 15–24	20	0.6	33.8 | < 0.001	0.45–0.89	27	0.9	45.97 | < 0.001	0.66–1.18
15–19	7	0.5	10. 05 | 0.002	0.28–1.00	12	0.9	16.30 | 0.001	0.58–1.54
20–24	13	0.7	21.50 | < 0.001	0.47–1.10	15	0.8	26. 14 | < 0.001	0.56–1.23
Airtime								
Total 15–24	77	2.4	109.21 | < 0.001	2.00–2.86	100	3.1	148.54 | < 0.001	2.68–3.68
15–19	33	2.4	55.18 | < 0.001	1.83–3.11	42	3.1	70.88 | < 0.001	2.47–3.96
20–24	44	2.4	58.70 | < 0.001	1.82–3.05	58	3.1	81.05 | < 0.001	2.54–3.91
Cellphone								
Total 15–24	43	1.4	51.26 | < 0.001	1.04–1.80	56	1.8	64.27 | < 0.001	1.38–2.24
15–19	12	0.9	19.65 | < 0.001	0.55–1.32	22	1.6	32.86 | < 0.001	1.11–2.18
20–24	31	1.8	38.52 | < 0.001	1.29–2.43	34	1.9	37.43 | < 0.001	1.318–2.63
Items for children or family								
Total 15–24	14	0.4	26.30 | < 0.001	0.28–0.61	25	0.8	36.68 | < 0.001	0.58–1.12
15–19	3	0.	5.04 | 0.025	0.09–0.50	10	0.8	13.06 | 0.001	0.45–1.33
20–24	11	0.6	20.27 | < 0.001	0.37–0.89	15	0.8	24.75 | < 0.001	0.56–1.24
Cosmetics								
Total 15–24	53	1.6	61.87 | < 0.001	1.26–2.07	81	2.4	103.12 | < 0.001	1.99–2.94
15–19	14	1.0	23. 03 | < 0.00	0.64–1.46	26	1.8	44.10 | < 0.001	1.35–2.45
20–24	39	2.1	47.18 | < 0.001	1.59–2.80	55	2.9	70.00 | < 0.001	2.27–3.65
Other								
Total 15–24	44	1.6	67.62 | < 0.001	1.27–2.4	17	0.7	27.97 | < 0.001	0.45–0.94
15–19	21	1.8	32.36 | < 0.001	1.25–2.50	8	0.8	11.38 | 0.001	0.42–1.36
20–24	23	1.5	29.76 | < 0.001	1.04–2.14	9	0.6	13.89 | 0.001	0.33–0.96

#### Socio–Economic Variables

Among sexually active survey participants, 19.4% reported that themselves or another household member went a day and night without eating because of lack of food (higher food insecurity), and 45.7% reported that their household depended on social grants (child support, foster care, disability grants, or a pension) (Table 2). Among sexually active AGYW, transactional sex was reported by significantly more of those who reported higher food insecurity (18.2%) compared with those who did not report higher food insecurity (10.7%; χ^2^ = 25.7, p < 0.001) (Table 2). Transactional sex was reported by 12.9% of AGYW who reported their household was χon a social grant, and by 11.5% of those who reported their household was not dependent on a social grant (χ^2^ = 1.8, p = 0.17) (Table 2).

Among sexually active AGYW, transactional relationships were reported by significantly more (21.9%) of those who reported higher food insecurity, compared to 11.7% of those who did not report high food insecurity (χ^2^ = 36.7, p < 0.001) (Table 3). Transactional relationships were reported by 14.7% of sexually active AGYW who reported their household was dependent on a social grant, and by 12.8% of those who reported their household was not dependent on a social grant (χ^2^ = 2.9, p = 0.09) (Table 3).

Table 5 below shows the association between sociodemographic characteristics (independent variables) and transactional sex (dependent variable) among participants who had ever had sex, adjusted for potential confounders such as age of AGYW, the age of sexual partner, and previous transactional relationships. Almost all variables included in the regression model were significant except for two; being in school and living in the household dependent on social grant. AGYW who were between the ages of 20–24 years, reported higher food insecurity, had a high alcohol use, a sexual partner older than her by 5 years or more, and had had transactional relationship in the past were more likely to report having transaction sex (see Table 5).

**Table 5:** Factors associated with transactional sex behaviour among sexually active AGYW (n = 3009)

Variable	Transactional sex (dependent variable)	
	Adjusted odds ratios (aOR)	95% Confident intervals (CI), p-value
Age category		
15–19 (ref)	–	–
20–24	**1.01**	**0.81**–**1.24 | p = 0.97**
Currently in school		
No (ref)	–	–
Yes	1.0	0.82–1.19 | p = 0.91
Reported higher food insecurity		
No (ref)	–	–
Yes	**1.87**	**1.53**–**2.29| p** < **0.01**
Household social grant dependency		
No (ref)	–	–
Yes	1.06	0.88–1.27 | p = 052
Had high alcohol use (Audit-C score 2 or higher)		
No (ref)	–	–
Yes	**1.50**	**1.25**–**1.79 | p** < **0.01**
HIV status		
Positive (ref)	–	
Negative	**0.69**	**0.56**–**0.84 | p** < **0.01**
Had transactional relationship in the past		
No		
Yes	**61.1**	**47.37**–**78.76 | p** < **0.01**
Sexual partner older than 5 years		
No (ref)	**–**	**–**
Yes	**1.89**	**1.58**–**2.26 | p** < **0.01**

*Bold* significant

### Qualitative Findings

Among the 237 AGYW respondents aged 15–24 years in the qualitative sample, the mean age was 17 years. The 38 male respondents were aged between 18 and 23 years old, with a mean age of 19 years.

#### Motivations for Engaging in Transactional Sex and Relationships

##### Contexts of Poverty

Sharing perceptions of the reasons that AGYW engage in TSR, AGYW respondents from across the study sites described poverty and food insecurity as being a key driver: “(Transactional relationships/girls accepting money from men) happens because there is a situation that compels you to accept money… in households where you are poor or there is something you don’t have, it becomes easy to go to a certain boy to ask money so that you can feed your kids and family… you are selling yourself… not asking for help but selling your body” (MPU, AGYW, 15–19 years). Being bought basic food necessities by transactional partners was described: “my boyfriend is there for me… not that I want gifts and money and all that… (but) he buys me pap (maize porridge) and all those things” (NW, AGYW, 15–19 years). For some, TSR was viewed as a mechanism of ‘survival’: “Coming from a poor background… it is a way of trying to survive” (EC, AGYW, 15–19 years). The need to pay for costs related to education, including school/tuition fees, school uniforms, and stationary, was cited as a motivating factor for TSR for AGYW from poor families: “Some girls are doing it for pleasure, others are doing it in order to benefit… if you do not have money for school needs… then you end up selling your body to get money to get education” (MPU, AGYW, 15–19 years).

For AGYW who come from poor families, in a context of high unemployment rates and few opportunities for income generation, TSR is viewed as a legitimate means of attaining material and/or financial support: “What often leads us to boys, and ending up sleeping with them is because you are unemployed and you need money… your family home needs money. You end up going out there… your parents are not working and they have nothing. You end up throwing yourself at a boy, not because you love them… because you want money, because that boy supports you. We also end up throwing ourselves at sugar daddies because of such things” (WC, AGYW, 20–24 years).

The association between transactional relationships and age disparity between partners was highlighted: “Girls have intimate relationships with adults because they need money and they are given money” (MPU, AGYW, 15–19 years). For those AGYW with deceased parents who need to support siblings and family members, transactional relationships with ‘blessers’ (a commonly used South African colloquial term for an older man who provides a younger woman with material/financial support) offer an accessible source of income: “Girls are forced by the situation at home and fending for the family. They think it’s the way to go… If parents have passed on and you have to look after the family. You will then decide to fall in love with older people in order to get money” (MPU, AGYW, 20–24 years). For some AGYW, low self–esteem and the desire for affection, lead them to engage in transactional relationships: “When you have made peace with the fact that you won’t be loved… you resort to blessers” (EC, AGYW, 15–19 years).

##### Glamour, Luxury and Social Prestige

Importantly, not all AGYW who engage in transactional sex do so out of desperation, and a need for basic material necessities. Some AGYW shared their own experiences of doing so in order to buy the things they desire/‘want’: “We are from poor families or not well–to–do families… having an affair will help you get money and everything you want” (MPU, AGYW, 20–24 years). Besides food insecurity as a driver for transactional relationships, some AGYW desire to eat ‘luxury foods’: “You do it (enter into transactional relationship) because things at home are not good, maybe you are struggling to make ends meet… you will see that it’s better to go to the guy because… in the end you will have luxuries like Doritos… you are there for food” (KZN, AGYW, 15–19 years).

Qualitative narratives echoed the survey data showing that cosmetics and clothes were amongst the items most commonly exchanged in transactional sex or relationships: “I get money from my boyfriend… I use it to buy cosmetics (toiletries) and clothing… (there are benefits to having a boyfriend)… the little money that he gives me… is a good thing… money to buy clothes… it is a very common thing for a girl to date a man because she wants money for clothes (KZN, AGYW, 15–19 years). Social status attached to hairstyles was also evident in the AGYW narratives, with money for new hair extensions being an incentive to engage in TSR: “I have a boyfriend… he takes care of me… he buys me weaves (hair extensions)” (NW, AGYW, 20–24 years).

The desire for ‘luxury’ and ‘glamour’ was one of the key themes that emerged in AGYW narratives around transactional relationships, particularly with older partners: “It is a common practice for teenagers to fall in love with older people because they like glamour. These older people do things for them… girls fall for those who have cars… they even fall for the truck drivers because of money” (MPU, AGYW, 20–24 years). The desire for glamour is closely linked to social/peer pressure, to have what your friends have: “(When a friend) tells you about all the things she receives from this older person, you also want those things, like a weave (hair extensions). Girls are under peer pressure… following their friends” (EC, AGYW, 15–19 years). The prestige attached to wearing expensive branded clothing compared to the cheap and accessible clothing brands, leads some AGYW to engage in TSR: “We deal with peer pressure… you see your friend, she is beautiful, she is wearing labels (name brands) and… they are driving a Range Rover… your clothes are from PEP (cheap clothing store), you have to accept, but… it’s hard… So peer pressure (leads to)… exchanging sex for money” (NW, AGYW, 15–19 years).

The ability to eat out at restaurants, compared to having to eat staple foods such as maize porridge, was also regarded as a sign of prestige: “Girls go around, bragging, like ‘My friend you know what, yesterday we ate at Romeno (pizza restaurant)!’…Then if you were having pap (maize porridge), you think… yesterday I had pap… I also want to be like her, I also want to eat Romeno pizza, and then you have to go to that man, and he will be like ‘Ah baby, let’s go to Romeno’… and then… you get into trouble …only trying to be like others**”** (NW, AGYW, 15–19 years). The provision of alcohol and other substances by partners was also cited as a motivating factor to engage in TSR: “Girls fall in love with working boyfriends… they buy them liquor and drugs… they have money, and they end up drinking liquor. You find them strolling with different boyfriends (having multiple partners)” (MPU, AGYW, 20–24 years). Some AGYW become dependent on men buying alcohol for them: “There is a bar nearby (to where I work). When I arrive in the morning there are girls just sitting there… They say ‘we are waiting for anyone who will come and buy for us… give us some alcohol because we are thirsty, we’ve been waiting for sunrise’ [laughing]… they are always there in the morning …waiting for any man who will come to buy them alcohol and they will cling to him (KZN, AGYW, 20–24 years).

Respondents suggested that some AGYW actively encourage each other to engage in TSR, suggesting that having a ‘blesser’, an older partner who provides material support is desirable, preferable to dating boys their own age: “Girls often say …‘my friend, we are supposed to have the blessers so that they will be able to support us, school children (boys the same age) are small, they do not have money’” (NW, AGYW, 15–19 years). The expectation in relationships with blessers is that sex is traded for being bought/given material goods: “If they buy you stuff you will be inclined to say yes when he wants sex” (EC, AGYW, 15–19 years).

Apparent in the narratives of AGYW respondents was a sense that transactional relationships are useful and legitimate sources of income, which should be exploited to the benefit of AGYW. “If I need money I get it from my boyfriend… He even buys me some cosmetics (toiletries)… My family know about it… they realise… because previously I had nothing but now they are wondering where do I get things?… In my view, it’s okay because I still do not have a job for now… My boyfriend knows that I don’t have money (so) he gives it to me… Whatever I want, if I need clothes and want to go somewhere, he is able to provide for me… he is the one who will pay for me, do my hair, and all of that” (KZN, AGYW, 20–24 years).

The expectation to be financially/materially supported by partners was evident in the narratives of AGYW, who suggested if these expectations are not met, there would be relationship conflict: “the day my boyfriend doesn’t give me money we will have a problem… he shouldn’t be too stingy… he should know that… without me even asking… I don’t want a person who will tell me ‘no wait, you’ll get the money on Friday’ do you understand?… I feel like if you have it now, give it to me now, instead of you telling me… I’ll get it on Friday” (NW, AGYW, 20–24 years). The suggestion was made that AGYW would be justified in cheating on boyfriends if they failed to meet expectations of financial provision: “that’s why we end up cheating… a stingy person… because if you’re not satisfied… what do you do?… go outside (have sex with someone else)? …that is why I would cheat” (NW, AGYW, 20–24 years).

Describing their own agency in engaging in TSR, some AGYW suggested that having a blesser is fun, exciting, and makes you feel like a “lady”: “Girls like to say, if you want to be a lady, for you to know what we feel, give him what he asks… have sex with the dude because it is fun, it is exciting. You don’t have to care what other people say” (NW, AGYW, 15–19 years). For some AGYW, having a blesser, or older partner who supports you financially, is a sign of independence, success and maturity, and something to aspire to: “She had a nice body… (so) she is able to get a partner. She then went to the tavern to get blessers… she said that as long as she has a blesser, it means that her life has started… There is no need to go to school, the future is the future for as long as she has a partner who has money and can support her… (She said) ‘Mmmm!… school is boring… I will get someone who will support me’” (NW, AGYW, 15–19 years).

#### Risks Associated with Transactional Sex and Relationships

The exchange of material/financial compensation for sex informs the unequal power dynamics in transactional relationships: “the person sponsoring (providing money) has more powers… because… he is paying… giving her his money… she has to do whatever he wants” (MPU, AGYW, 15–19 years). Related to power dynamics, AGYW suggested that transactional sex is more likely to be condomless, and thus lead to HIV infection and unintended pregnancies: “You can contract diseases like HIV/AIDS and STIs… A man as that (a blesser), he has much power because he provides… he gives you money, he does everything, so he wants you to submit to him” (MPU, AGYW, 15–19 years). AGYW voiced the view that with financial attainment being the priority and sole purpose of entering into transactional relationships, other issues, like their partner having multiple concurrent partnerships, are of little importance: “I would like someone with money [laughter]. When he gives me money and maintains me, I will be satisfied, I don’t care if he has other girlfriends, I just don’t care, as long as he gives me money” (KZN, AGYW, 15–19 years).

In cases where AGYW come from poor households, the power differentials, and likelihood of engaging in risk behaviours, are enhanced: “Because of the situation at home… growing up from disadvantaged backgrounds, us girls opt to date blessers and end up sleeping with them without protection… That’s how we fall pregnant” (WC, AGYW, 15–19 years). Respondents suggested that TSR resulting in pregnancies likely lead to abandonment: “A guy will give you money, then sleep with you and you become pregnant, then after having the child you ask for money, and he won’t give you money, giving an excuse that he won’t be able to maintain two people” (KZN, AGYW, 15–19 years). Likewise, those transactional relationships that result in HIV infection will also be ended: “You will find that you have contracted diseases and he will leave you afterwards” (MPU, AGYW, 20–24 years).

In contrast to the views expressed above which ‘normalise’ transactional sex and transactional relationships, suggesting that it is an acceptable strategy for attaining glamour and status, some of the AGYW respondents shared views that AGYW’s belief of having agency in these relationships was naïve: “You shouldn’t love a person because he has money… money means nothing… nowadays, a lot of people like so many things… (but) money doesn’t help you with anything because one might have money.. (but) they don’t love you, they are using you, so no, I don’t agree with this” (KZN, AGYW, 15–19 years). Respondents spoke of the lack of emotional connection or commitment in transactional relationships: “Blessers are not good at all because it’s not like he loves you or he wants to marry you. They just want to play you and give you everything so that you will think they love you, whereas they are using you” (MPU, AGYW, 20–24 years). Respondents spoke of the way in which older men/blessers take advantage of AGYW’s lust for glamour and luxury: “(Blessers) are not good at all because they take advantage of young girls. They know that we like things and money… they test you by asking you if you don’t want to be taken out. Because you are forward you will respond by saying yes and that is how it will begin” (EC, AGYW, 15–19 years).

Some of the AGYW respondents suggested that they would not be impressed by a partner offering material wealth, and that love is more important than being fashionable and glamourous: “I don’t want a person with money… for me it doesn’t matter for as long as we love each other. No need to flatter… or show off about your clothes… I am not impressed with that, a person must be simple, as long as we love each other, care for one another and forgive each other” (EC, AGYW, 20–24 years). One of the AGYW respondents commented on the importance of AGYW finding alternative means of support, instead of entering into TSR: “Even if she comes from a poor background she must not lose hope, the government will take care and help where they can… she does not have to sleep with people” (EC, AGYW, 15–19 years).

#### Male Views on Transactional Sex and Relationships

Male peer respondents in the qualitative study shared their views on AGYW being “gold diggers” (WC, Male Peer), engaging in TSR. One commonly held view was that AGYW are materialistic and only interested in money: “Girls nowadays love people with money. If you have money they will be after you” (EC, Male Peer); “Girls… love people with cars and money… they are after money” (EC, Male Peer). Related to this was the perception that AGYW are impressed by visible material wealth and money, as evidenced by physical appearance and clothing: “Girls are easily convinced, when you show them money, you dress smart, you look handsome, they agree to sleep with you… just like that” (MPU, Male Peer). Some of the male respondents shared their feelings that attaining material wealth is the only way to attract a female partner: “Why plan a future with a girl if you don’t even have money?… school is first, then you get the job, then you get a house and car, then you get the gift… with the girls… As soon as you get the car the girl is gonna follow” (EC, Male Peer).

Evident in the narratives of male respondents was the recognition of the normalcy of TSR, and the need to have sex with a partner in order to receive support: “Most girls get themselves into relationships because they need money or they need some other things, support… for the girl to get that thing, she must sleep with him” (KZN, Male Peer). Some of the male respondents explained that they prefer relationships that are transactional in nature as it puts them in a position of power and indispensability as the provider: “I prefer a girl who asks for money… because the girl who is self–sustained (doesn’t need your money), might leave you, she doesn’t feel like she needs you, she’ll leave. Whereas the one who asks for money, she might love you, even though it’s for the money, she loves you for the money, she still needs you” (NW, Male Peer). However, not all young men appreciate the accepted expectation of the transactional nature of relationships: “I feel used because it means she doesn’t love me for love, she loves me for money, she’s using me” (NW, Male Peer).

Male respondents described their lack of respect for AGYW who engage in TSR, especially those who exchange alcohol for sex: “Girls should be respecting themselves so that I as a male can respect them as well. I can’t respect them if I buy beer for them, and now they come and drink my beer… guys use that as advantage to have sex with girls. They buy beer and girls come drink the beer, after they must go to a private place… they will go and have sex… I want the girl who can respect herself so that I can do the same for her” (EC, Male Peer); “A girl should learn to think for herself, you cannot sleep with a guy just for a beer… He won’t even give you money, he will buy you a few beers, sleep with you and leave you, that is how we operate as guys” (MPU, Male Peer). Male respondents also described AGYW who engage in transactional relationships with older men as impure and sullied: “When we date them they are already damaged goods, dating older men… yet they are the same age as us” (EC, Male Peer). Young men also felt that their own risk of HIV infection is increased as AGYW their age are involved with older men/ “blessers”, at the same time as being in relationships with boys their own age.

## Discussion

Analysis uncovered complex motivations for engaging in transactional sex and transactional relationships, ranging from economic deprivation, hunger and poverty, to desires for social approval, prestige, glamour, and luxury. Narratives revealed prevalent socio-cultural norms informing the way in which TSR are perceived in these communities, including the normalisation of TSR amongst AGYW, and the practices of using sex as a currency for exchange. With survey data revealing that money was the commodity most commonly exchanged for sex, and the most frequently endorsed response option for reason for starting or staying in relationships, the boundaries between ‘commercial sex work’ and ‘transactional sex/relationships’ were shown to be unclear, subjective and context dependent.

In the qualitative data, poverty and food insecurity were listed as key drivers for AGYW engaging in TSR. Those AGYW from poor families, who have to provide support to siblings and family members, and with few opportunities for income generation, regarded TSR as a valid means of attaining material and/or financial support. Respondents suggested that the need to pay for basic costs related to food, education, and clothing led more economically vulnerable AGYW to engage in TSR. Survey reporting of TSR was higher among AGYW who reported experiencing higher food insecurity compared with those who did not.

However, AGYW motivations to engage in TSR cannot be reduced to poverty related factors. In our study, both AGYW and male respondents described the way in which TSR are closely associated with materialism, and a desire for glamour and luxury, among AGYW in South Africa today. It is possible that for AGYW who come from poor families and resource constrained settings, the desire for visible material wealth, and the prioritisation of ‘bling’ and material prestige, influence AGYW’s relationship choices and act as contributory factors in the decision to engage in TSR. The desire that our respondents expressed to wear labelled clothing and eat at restaurants, is framed within a broader social prioritisation of visible material wealth and social approval. Finding a male partner who is able to provide status–inferred expensive commodities may be part of AGYW’s desire for what has been termed 'symbol capital', in this case symbols of a modern and successful life [11, 21].

In the survey, clothes and cosmetics/make–up were among the most commonly listed items that AGYW reported entering into a transactional relationship for, after money and airtime. We divided our qualitative findings into items that AGYW “want” versus those that they “need” for basic survival. For example, where our respondents spoke of poor households and families, and the need to buy food and pay education costs, we categorised this as “need”. Where AGYW spoke of the desire for glamour, hairstyles, branded clothing and restaurant meals, we categorised this as “want”. Prior research has all used framing of items for ‘survival’ (need) versus ‘consumption’ (want) [22]. These categorisations have also been problematised for the reason that researchers, not AGYW themselves, have constructed these hierarchical/binary classifications,constructs of luxury and necessity are subjective and context dependent [22]. In addition, while these categories might be useful to frame ‘wants’ as those items non–essential for survival, it may be true that they are “needed” as key ingredients which confer peer approval, social status. For example, fashionable, branded clothing may be deemed ‘essential’ for preventing social exclusion, which for adolescents and young people, who tend to be highly sensitive to how their physical appearance is perceived by peers, and therefore essential for mental health and self–esteem [22, 23]. Additionally, clothing and outward appearances are mechanisms through which young people express their identities, and their ability to portray themselves in a ‘socially–acceptable’ manner may be intricately tied up with self–esteem, which itself is linked with vulnerability to sexual risk [22]. Lastly, when researchers engage in the hierarchical categorisation of items for which AGYW exchange sex, there is a danger of misclassifying items that AGYW consider important needs as frivolous luxuries [22]. A consequence of this misinterpretation could be that interventions are inappropriately designed according to a misconstrual of the true underlying motivations of AGYW sexual risk behaviour [22].

Some AGYW respondents commented that transactional relationships with blessers can be exciting and engender a feeling of adult maturity and independence. The sense of self–worth and power that AGYW gain from exploiting their sexuality in exchange to gain material assets may explain why many AGYW engage in TSR even when their basic material needs are provided for by family members [24]. Attaining financial independence has been identified as an important driver of young women entering into transactional relationships [3]. Given that so few of the AGYW respondents who were out of school were employed, it is possible that transactional relationships were considered a valid source of income. Research has suggested that in settings with high unemployment where there is a lack of alternative sources of income, examples set for AGYW by older sister or friends, demonstrate that one means of acquiring material goods, and paying for “non–necessary” items that family spending budgets do not allow, is through transactional relationships [3, 14].

As our respondents described, peer pressure to conform to these standards was a motivating factor in AGYW engaging in TSR. Using transactional sex as a means of acquiring the material possessions needed to belong and attain peer respect and approval has been found in previous research among young people [3, 7]. In a social and economic context where AGYW’s feelings of self–worth and self–esteem are closely associated with their appearance and material possessions, having a means of attaining these items is a pathway for developing their own identity within their peer networks [14].

Links between TSR and alcohol use emerged in our findings. In the survey, reporting of TSR was higher among AGYW who had high alcohol use and alcohol use was strongly associated with transactional sex in the regression analysis. In the qualitative data, male respondents described their perceptions that it is a common practice for AGYW to have sex with men who purchase alcoholic drinks for them. Male respondents in our study suggested that AGYW should be more sensible, and take care of themselves, as men’s behaviour of buying alcohol in expectation of receiving sex in exchange, is unlikely to change. Findings from previous ethnographic research conducted in Cape Town corroborate our respondents’ assertions around the commonality of alcohol being exchanged for sex, suggesting young women who frequent shebeens (township bars) do so with the expressed intention of finding men to pay for their drinks and that sexual encounters usually follow [25–27]. Links between transactional sex and alcohol consumption have previously been described, suggesting that accepted social norms determine that if a woman accepts drinks bought by a man, she should expect to have sex with him in exchange [26–28]. Men use alcohol as a currency with which to trade for sex with women,in these transactions the paying man holds the power in negotiating the conditions of sex, including condom use [26]. Evidence also suggests higher HIV risk among AGYW who frequent shebeens [25].

Evident in the narratives of male respondents in our study was the recognition of the perceived normalcy of TSR, and the notion of men as material/financial providers, in exchange for sex. Some of our male respondents explained their preference for relationships that are transactional in nature as it puts them in a position of power and indispensability as the provider. Transactions related to sex have been described as an embedded expectation in heterosexual romantic relationships in South Africa [12, 29]. Our findings support the notion that men’s role as providers is linked to masculine identities, and young men’s sense of self–esteem [2]. Socioculturally scripted gender roles in sub–Saharan Africa frame men as providers and women as receivers, who expect to be materially supported by male partners [4, 23]. The notion of women as financially dependent on male partners appears to be socially normative in sub–Saharan Africa, and one of the key structural drivers of gendered socio–economic power disparities in the region [6]. The belief held by South African men that sex does not come for free but must be exchanged for something demonstrates the commodification of female sexuality [24]. These social norms framing gendered power discourse serve to reproduce certain unequal gendered power structures and gender role expectations [23, 30]. Material/financial provision by males can be viewed as the mechanism through which they attract and control female partners, and hold the reins in sexual decision making [30].

Survey data revealed an association between HIV positive status and reporting of transactional sex among AGYW in our sample, with HIV positive AGYW more likely to report transactional sex experiences than HIV negative AGYW. In the qualitative data, AGYW respondents described the way in which the exchange of material/financial compensation for sex informs the power dynamics in transactional relationships. AGYW suggested that because of power disparities between partners, transactional sex is more likely to be condomless, and thus lead to HIV infection and unintended pregnancies. While AGYW may feel a sense of power and actively seek multiple partners to meet their needs or desires for material support, at the same time, the unequal power dynamics inherent in these economically asymmetric relationships enhances their risk [31]. Men, as the financial providers, are entitled to dictate sexual interactions [23]. Evidence corroborates the findings that gendered socio–economic power disparities are a key driver for AGYW’s vulnerability to HIV infection, and that participation in transactional sex is associated with condomless sex and HIV seropositivity among AGYW in South Africa [2, 31].

With regards to the links between TSR and HIV risk, comments made by AGYW in our study suggest that those AGYW who engage in TSR are likely to have multiple partners, depending on the current financial status and availability of partners. This finding concurs with the conceptualisation of multiple sexual partners as being on the pathway between TSR and HIV [12]. Additionally, young men in our study shared perceptions that AGYW who engage in transactional relationships with older men are ‘damaged goods’, likely to have had sex with multiple partners. Links can be made between this narrative and evidence suggesting a positive association between age–disparate partnerships and HIV–infection risk among young women [10, 12]. AGYW with older male partners have been found to be more likely to practice other behaviours that exacerbate their HIV risk, including having a greater number of partners, engaging in transactional sex, and having condomless sex [36]. Due to the heightened power imbalances in age-disparate partnerships, AGYW are less likely to be successful in negotiating condom use with older partners [12, 13]. An important distinction to make however is that whilst TSR are often characterized by age-disparate partnerships, age-disparate partnerships are not always transactional in nature [16].

In addition these is an association between TSR and multiple concurrent sexual partnerships, exacerbating HIV risks. As our respondents suggested, AGYW may have concurrent relationships with ‘blessers’ (financial providers), at the same time as romantic relationships with boyfriends of their own age. It has been demonstrated that AGYW who report staying in relationships for economic reasons, or engaging in transactional sex with non–regular partners, are more likely to have multiple concurrent sex partners [31]. Maintaining multiple concurrent relationships enables AGYW to fulfil their different needs and desires, providing romantic/emotional connection, at the same time as receiving material support [4, 31].

Researchers have problematised the assumption that TSR is necessarily based on poverty, with young African women as the victims of male sexual and financial power [32]. It is evident that some AGYW are active social agents in these relationships, realising the economic potential of their sexuality and cognisant of the personal gains to be made, they exploit men’s lust to uplift themselves and attain their material aspirations [8, 21, 23]. That young women exercise their autonomy in actively and strategically engaging in such relationships challenges the idea that young women are passive, hapless victims of predatory older men [24, 32]. Indeed it has been suggested women are aware of their own agency and power in these transactions, and use explicit strategies to exploit and extract resources from male partners, as well as having control over the initiation, maintenance and termination of relationships [26, 33]. However, there is a need to recognise the reality of the South African context and gender inequity in which AGYW have reduced access to economic resources [32]. This gendered economic inequity places South African men in an economically privileged position, framing notions of masculinity as being linked to control over economic resources, referred to as ‘provider masculinity’ [32]. Within broader macro socio–economic contexts where there are high levels of unemployment, and AGYW have limited access to income generating activities, sexual attractiveness and desirability become valuable resources [34].

Previous literature has outlined three paradigms with which to organise and explain motivations for transactional sex: (1) sex for basic needs; (2) sex for upward mobility and status, and (3) sex for material expressions of love [3]. Findings from the analysis of qualitative data in our study primarily related to the first two. In recognising the complexity of motivations for engaging in TSR, and the heterogeneity of such interactions it is important to allow that motivations are not mutually exclusive and often AGYW are driven by a combination of subsistence needs and consumerist desires [14, 23]. The decisions that AGYW make about entering into such relationships may be based on various reasons that differ in relation to specific contexts [23]. It is therefore critical to take into consideration both the vulnerability and agency of AGYW in the specific context that shapes their choices [23].

Several limitations of our study should be stated. (1) Data presented comprise a subset from a larger data set from a study (secondary data analysis) in which the examination of TSR was not a key focus of inquiry; although both quantitative and qualitative data on TSR were collected, limited detail was available. (2) Although the overall sample realisation rate for the survey was comparative with sample realization rates of the 2016 South African Demographic and Health Survey (SADHS), and the South African National HIV Prevalence, Incidence, Behaviour and Communication Survey, 2017 (HSRC, 2019), it was relatively low. (3) Given the cross-sectional design of the survey, it is not possible to determine whether HIV was an antecedent to or consequence of TSR, and the same applies to higher food insecurity and alcohol use. (4) The survey did not directly ask about sex in exchange for alcohol or substances, and the data lacks event-level detail on how individual episodes of drinking are associated with sex and sexual risk in TSR. (5) Both quantitative and qualitative study components relied on participants’ self–reports. Since TSR may be considered undesirable or socially stigmatised behaviours, it is possible that there was under–reporting, as AGYW who engage in transactional sex seldom disclose that they have exchanged sex for money [25]. (6) We employed the terms “transactional sex” and “transactional relationships” in this study, however we did not investigate participants’ own understandings and conceptualisations of the definitions and terminology related to transactional sex or sex work. (7) Our data on perceptions of HIV risk amongst AGYW engaging in TSR is limited,as TSR was not an initial focus of the inquiry, there was limited in-depth probing around the topic at the time of data collection. (8) Limitations in the qualitative sampling relate to this study being part of a larger intervention evaluation study, and thus the selection of respondents was not determined by reporting of TSR; this is likely to have impacted on the views represented. Additionally, older men who engage in TSR with AGYW were not included in the sample; this population are hard to engage in research, and may be unlikely to speak openly about TSR, but their perspectives would add great value to understanding the dynamics of TSR. (9) Lastly, a weakness in this study which should be noted relates to the limited measurement of household food insecurity; the complexity of which is has been previously described [35].

### Conclusions and Recommendations

Interventions need to encourage AGYW to critically reflect on their own agency and choices in transactional sex and relationships, their aspirations for consumer items that symbolise a better life as motivation for sexual exchange, and the norms and beliefs that sustain gender inequality in transactional sex relationships [16]. Instead of simplistic framing of TSR as inherently risky and wrong, interventions need to consider the driving motivations, and the realities of negotiating the complex dilemmas of risk versus gain, helping AGYW to identify and navigate risks safely, and protect themselves, while maintaining some of the benefits [8]. Young men and women also need to be encouraged to reflect on relationship and sexual values, and the gendered expectations of male provision and what it “buys” men in return [7, 8]. ‘Gender transformative interventions’ that aim to critically address shared societal expectations that women should have sex with men in return for their material/financial support, and work to challenge provider norms, masculinity and the concept of control of women in heterosexual relationships, should also be combined with economic empowerment interventions for AGYW that may help to reduce the extent to which AGYW need to rely on male providers [29, 30]. Importantly, Wamoyi and Stoebenau (2018) make the recommendation that instead of interventions trying to address the practice of transactional sex itself, they should rather try to address the associated HIV risks, and integrate measures into broader empowerment and health interventions, rather than attempt to intervene on transactional sex alone. Another important aspect to include in interventions relates to age-disparate partnerships; efforts should be made to engage AGYW and older men in order to build skills in critical reflection on the short-, medium- and longer-term benefits and costs of engaging in age-disparate sexual relationships [16].

Considering the range of motivations for AGYW to engage in transactional sex and relationships in these communities in South Africa, and the associated HIV risks, addressing these behaviours is critical as part of South Africa’s HIV response. Our findings help build the evidence base with which to inform the design of contextually appropriate interventions to address sexual risk among AGYW in South Africa.

## Data Availability

The dataset used for the current study is available from the corresponding author on reasonable request.
